# Neutron Scattering at the Intersection of Heart Health Science and Biophysics

**DOI:** 10.3390/jcdd2020125

**Published:** 2015-06-02

**Authors:** Drew Marquardt, Richard J. Alsop, Maikel C. Rheinstädter, Thad A. Harroun

**Affiliations:** 1 Institute of Molecular Biosciences, Biophysics Division, University of Graz (NAWI Graz), mbox Graz 8010, Austria; 2 BioTechMed-Graz, Graz 8010, Austria; 3 Department of Physics, Brock University, St. Catharines, ON L2S 3A1, Canada; E-Mail: thad.harroun@brocku.ca; 4 Department of Physics and Astronomy, McMaster University, Hamilton, ON L8S 4M1, Canada; E-Mails: alsoprj@mcmaster.ca (R.J.A.); rheinstadter@mcmaster.ca (M.C.R.); 5 Canadian Neutron Beam Centre, Chalk River, ON K0J 1P0, Canada

**Keywords:** neutron scattering, aspirin, cholesterol, vitamin E, Cardiovascular diseases

## Abstract

There is an urgent quest for improved heart health. Here, we review how neutron radiation can provide insight into the molecular basis of heart health. Lower cholesterol, a daily intake of aspirin and supplemental vitamin E are argued to all improve heart health. However, the mechanisms behind these common regimens, and others, are not entirely understood. It is not clear why a daily intake of aspirin can help some people with heart disease, and the benefits of vitamin E in the treatment of reperfusion injury have been heavily debated. The molecular impact of cholesterol in the body is still a hot topic. Neutron scattering experiments present a unique opportunity for biophysicists attempting to address these problems. We review some recently published studies that are advancing our understanding of how cholesterol, vitamin E and aspirin work at the molecular level, by studying the impact of these molecules on the cell membrane. These insights engage the broader health science community with new ways of thinking about these molecules.

## 1. Introduction

The molecular mechanism by which drugs and nutrients interact with the membranes of our cells is a central issue in pharmacological sciences. Cellular membranes are complex assemblies that are much more than simple permeable barriers or passive substrates for proteins [1]. Rather, they play an active role in many cellular functions, and they have a rich metabolism of their own. Many of these functions rely on a diverse array of lipids, vitamins, sterols, proteins and carbohydrates [2,3,4]. Vascular diseases are the leading cause of death in adults. Of the six types of cardiovascular diseases highlighted by Health Canada, ischemic heart disease is the leading cause of death, accounting for 54% of all cardiovascular deaths Mortality, Summary List of Causes 2008, Catalogue No. 84F0209X, Statistics Canada) [5]. Ischemic heart disease occurs when the blood supply to the heart muscle (myocardium) is cut off. Commonly, ischemia is a result of the accumulation of cholesterol-rich plaques in the coronary arteries (atherosclerosis).

The blockage of blood flow is not the only life-threatening condition that arises from ischemia. When treated, the restoration of the blood supply (reperfusion) can cause further damage to the myocardium, through oxidative stress, specifically free radical damage. The damage done during blood restoration is known as ischemia-reperfusion injury and also occurs during surgery when blood vessels are cross-clamped [6]. Ischemia-reperfusion injury has been extensively studied, but the underlying molecular mechanisms of the pathology and treatments remain a mystery [7]. Below, we discuss the role of neutron beams in the understanding of three small molecules with significant implications in the cause (cholesterol), preventative measure (aspirin) and recovery (vitamin E) of myocardium ischemia and reperfusion injury. The availability of neutron beams is crucial to obtain molecular-level information relevant to these molecules.

## 2. Neutron Scattering

The study of membranes and membrane-bound molecules requires precise conditions, such as temperature, pressure and hydration, to mimic physiological conditions. Neutrons provide a valuable method for examining these systems *in-vitro*, because certain materials (*i.e.*, aluminium) are “invisible” to neutrons, allowing for sample environments to be easily constructed and implemented [8]. Neutron scattering studies of biologically-relevant samples have an intrinsic advantage due to the abundance of hydrogen (${}^{1}$H) atoms that can be replaced (labelled) by deuterium (${}^{2}$H). The substitution of deuterium atoms for hydrogen, at selective locations, provides a contrast between two different samples (“labelled” and “unlabelled”). The difference in scattering length density between the labelled and unlabelled sample yields the location and distribution of the ${}^{2}$H label [9,10]. Gordeliy and Chernov theoretically determined that the spacial resolution of a functional group can be improved from the canonical resolution of $d/h_{max}$ (d∼ 50 Å, $h_{max}$ = 5) down to 1 Å by means of ${}^{2}$H labelling [11]. This ability to increase resolution by means of isotope substitution is an advantage to the more accessible X-ray scattering.

At continuous, reactor sources, collimated, monochromatic beams are typically used to obtain a good *q*-resolution and, with it, good spatial resolution. Neutron spallation sources use broad wavelength bands and time of flight techniques to receive high resolution. The scattered beam is measured by a detector (punctual, mono- or bi-dimensional) and the intensity of the outgoing over that of the incoming beams recorded as a function of the wave vector transfer, *q*. The angle between the incoming and outgoing beams is defined as the scattering angle, 2θ. The scattering vector, *q*, is then given by q=4πsin(θ)/λ. Because only the intensities of the scattered waves can be measured and not the phase shifts, a direct transformation back into real space is usually inhibited.

### 2.1. Contrast Variation

**Figure 1:**
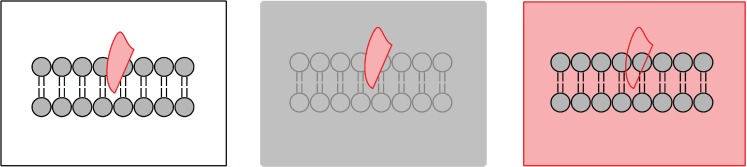
Schematic of possible neutron contrast variation experiments for a membrane (grey) with a protein inserted (pink). The left diagram represents the system with no contrast matching. The protein is highlighted in the centre diagram when the solvent (water) is contrast matched to the lipid bilayer. Membrane properties can be studied from the diagram on the right when the solvent is contrast matched to the protein.

**Figure 2:**
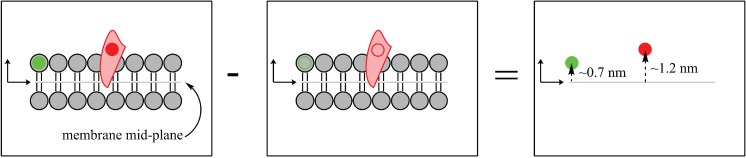
Biological systems have an intrinsic ability to be labelled due to the abundance of hydrogen (${}^{1}$H) atoms that can be replaced (labelled) by deuterium (${}^{2}$H). The substitution of deuterium atoms for hydrogen, at selective locations, provides a contrast between the “labelled” sample (left) and the “unlabelled” sample (middle). The difference in scattering length density between the labelled and unlabelled sample yields the precise location and distribution of the ${}^{2}$H label (red circle, right).

Compared to other biophysical techniques, neutron beams may offer distinct advantages when studying biological systems at the atomic level. Neutron scattering does not rely on bulky fluorophore or spin label probes, which can alter the physical properties of model membrane systems [10,12]. Instead, neutrons scatter off the nucleus of the atom, with the greatest scattering power coming from light elements commonly found in biological systems, such as carbon, hydrogen, oxygen and nitrogen. Contrast may be manipulated systematically by substituting an element with its isotope, as neutrons scatter differently from different isotopes. The substitution of hydrogen for deuterium is commonly used to manipulate contrast, as shown in Figure 1 and Figure 2 [13]. Scattering from individual components of the system, such as lipid, solvent or protein, can be suppressed through contrast matching with solvent, allowing for robust determination of bilayer structural properties, such as bilayer thickness, organization and composition, as shown in Figure 2.

### 2.2. Elastic Neutron Scattering

Crystallography is most useful and provides the highest resolution structures when the molecules are organized in a regular, crystalline environment. However, biological materials, in particular under physiological conditions, are inherently disordered and highly dynamic in nature. Fluctuations on different coherence lengths may lead to the formation of static or dynamic patches, so-called rafts. The challenge is to get the most amount of structural information out of this disordered, dynamic state of matter. Neutron diffraction has become a standard technique in biology and “life sciences”, which are considered as the key sciences of the twenty-first century. High-tech life sciences include the emerging biotechnology and biomedical device industries, functional foods and nutraceuticals, as well as the development of new biomaterials and pharmaceuticals.

The elastic X-ray and neutron scattering techniques, which were mostly employed in the past for membrane studies, are small angle scattering (SAS), small and wide angle diffraction and specular reflectivity. SAS is used to gain information on the shape, size and interactions of lipid lamellar systems and vesicles: it gives radially-averaged information in all directions of space. Small angle scattering experiments provide information about the size, shape and orientation of the different components of a sample for distances ranging from 50 to 5000 Å. Diffraction experiments determine the in-plane and normal structure of stacked lipid lamellar systems and can, for instance, locate an object within the lipid bilayer, such as cholesterol or peptides. Reflectivity is used to study the structure of bilayers in the direction perpendicular to the plane of the layers in a planar configuration and allows the determination of the structure and composition of a membrane with a very high spatial resolution [14].

The coherent scattering of neutrons provides information from the spatial correlation of nuclei, which is contained in the amplitude of the scattered neutron wave called the form factor (F(q)). F(q) is the sum of the coherent scattering length ($b_{coh}$, unique to each nucleus) of all of the atoms in the sample and is proportional to the measured intensity of scattered neutrons. The real-space distribution of scattering lengths (*ρ*, scattering length density) is the Fourier transform of the form factor. In the study of oriented model membrane structure and label location, the scattering intensity is detected when the Bragg condition is satisfied (neutron diffraction). The relationship between the scattering angle (*θ*) and the unit cell length (*d*) is expressed by Equation (1), where *λ* is the wavelength of the incident neutron, *h* is an integer (Bragg order) and *d* includes the thickness of both leaflets of the lipid bilayer and one full water layer. Each Bragg peak corresponds to a discrete point on point of the form factor.

(1) hλ=2dsin(θ)

A lower resolution, but arguably more biologically-relevant approach to the structural study of model membranes is small angle neutron scattering (SANS). SANS is a structural techniques capable of of studying model membranes in solution under biologically-relevant conditions. Like diffraction, it relies on the constructive interference of coherently-scattered neutrons. In the case of model membrane vesicles composed of a single bilayer (unilamellar vesicles, ULV), the scattering intensity is directly proportional to the F(q) [15]. The real-space distribution of nuclei in this case is modelled using a variety of strategies, keeping the contrast condition in mind [16,17,18].

### 2.3. Dynamic Neutron Scattering

**Figure 3:**
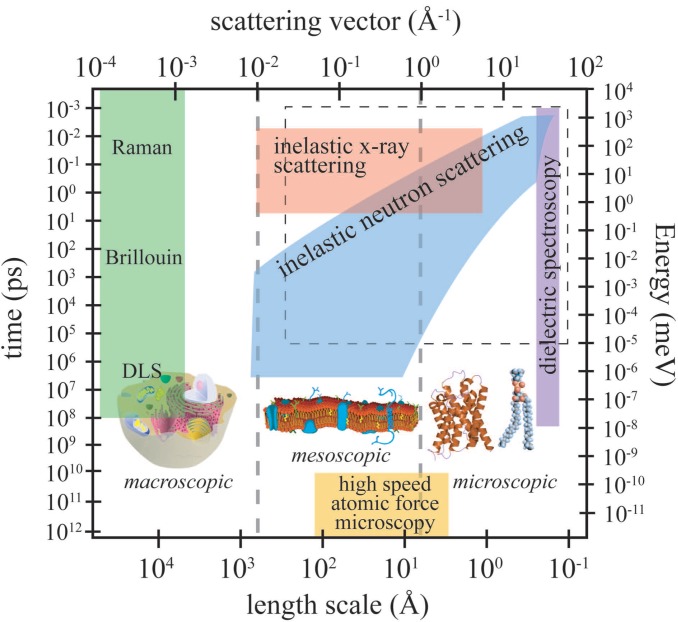
Accessible length and time scales and corresponding energy and momentum transfer, for some spectroscopic techniques covering microscopic to macroscopic dynamics. Light scattering techniques include Raman, Brillouin and dynamic light scattering (DLS). Inelastic X-ray and neutron scattering access dynamics on Angstrom and nanometre length scales. Dielectric spectroscopy probes the length scale of an elementary molecular electric dipole, which can be estimated by the bond length of a C–O bond (about 140 picometres). The area marked by the dashed box is the dynamical range accessible by computer simulations. High-speed atomic force microscopy is an emerging technique, which allows imaging in real space (adapted from [24]).

Biologically-relevant materials can be thought of as “multi-scale” materials, due to the fact that relevant dynamics take place over extended length and time scales [19,20,21,22,23]. To address this multi-scale behaviour experimentally, different techniques must be applied. Figure 3 depicts the length and time scales accessible by high-speed atomic force microscopy (AFM), inelastic neutron scattering, inelastic X-ray scattering, dynamic light scattering (DLS), Brillouin and Raman scattering and dielectric spectroscopy.

The relevant length scale for dielectric spectroscopy is in the order of an elementary molecular electric dipole, which can be estimated by the bond length of a C–O bond (about 140 picometres), and frequencies from kilohertz to gigahertz can be measured. Because the wavelength of the probe is usually around 510 nm or 632 nm, light scattering techniques are limited to small momentum transfers of about 10${}^{-4}$ Å${}^{-1}$ to 10${}^{-3}$ Å${}^{-1}$, corresponding to a length scale of about 100 nm. Inelastic neutron and X-ray scattering access length scales from smaller than Angstrom to more than 100 nm and time scales from picoseconds to almost one microsecond. High-speed AFM has combined a high spatial resolution of about 5 Å with a time resolution of milliseconds. In recent years, molecular dynamics (MD) simulations have proven to be an invaluable tool in developing models for molecular structure and dynamics in membranes and proteins. Because of the ever-increasing computing power and optimized algorithms, large complex systems (*i.e.*, many hundreds of molecules) and long simulation times can now be addressed. The dashed rectangle in Figure 1 marks the dynamic range currently accessed by computer simulations; the elementary time scale for simulations is in the order of femtoseconds.

In contrast to other spectroscopic techniques, inelastic neutron scattering results in wave vector-resolved access to molecular dynamics. For example, excitation frequencies and relaxation rates are measured at the different internal length scales of the system. A typical dynamic scattering experiment measures (*q*, ℏω) pairs, resulting in a frequency along with a corresponding length scale and possibly a corresponding direction, such as parallel or perpendicular to a protein’s axis. This additional information is of paramount importance when it comes to relating dynamical information to structure. In short, the suite of inelastic instruments used to study soft and biologically-relevant materials comprises time-of-flight, backscattering, triple-axis and spin-echo spectrometers [21,25].

## 3. Cholesterol

In mammalian cells, as much as 90% of all cholesterol can be found in the plasma membrane [26]. Cholesterol has been well established as a mediator of cell membrane fluidity. By interacting with lipid tails, cholesterol’s rigid structure causes the membrane tails to be constrained, thereby reducing membrane fluidity and altering potential lipid-peptide interactions [27].

**Figure 4:**
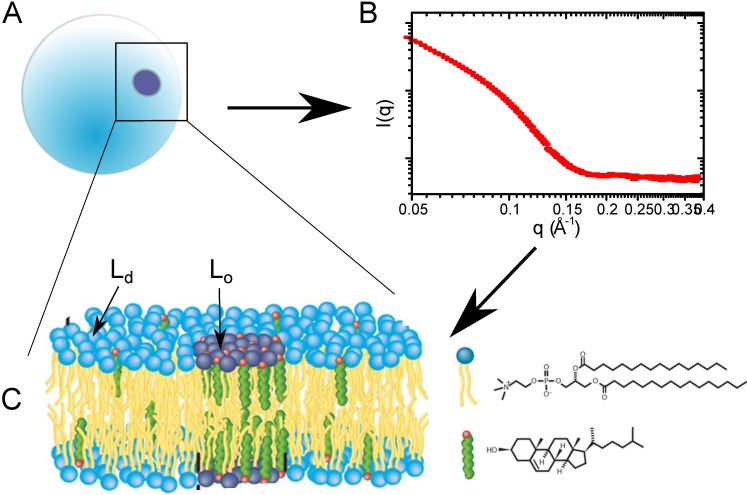
(**A**) Lipid vesicle containing $L_{o}$ domains; (**B**) small angle neutron scattering (SANS) curve, which contains structural information about the bilayer, as well as bilayer organization; (**C**) schematic of the membrane information contained within the SANS curve.

The action of cholesterol’s membrane mediation is observed through the formation of highly-ordered domains (so-called rafts) of model membrane areas enriched with cholesterol, as depicted in Figure 4. Interestingly, these domains are a unique lipid phase, which requires the presence of cholesterol, the liquid ordered phase ($L_{o}$). The cholesterol-poor counterpart to $L_{o}$ is the thinner and more disordered liquid disordered ($L_{d}$) phase [28]. At high concentrations of cholesterol, immiscible cholesterol bilayers may form [29,30]. These cholesterol “plaques” often occur in people with elevated cholesterol and play a role in diseases, such as atherosclerosis [31].

A study of nano-sized domain formation in free-floating bilayers was conducted by Heberle *et al.* by small angle neutron scattering. In order to mimic a complex biological membrane, this pioneering study examined four-component model systems containing a saturated phospholipid, varying ratios of mono- and di-unsaturated phospholipid and a constant cholesterol concentration for the presence of domains [32]. Domain sizes were found to increase with unsaturation (di-unsaturation:mono-unsaturation ratio), but more interestingly, there is a direct correlation between the domain size and the bilayer thickness mismatch of $L_{d}$ and $L_{o}$. This experiment used probe-free methods to observe these cholesterol-rich nanodomains and demonstrated how functional domains in cells may be regulated through changes in phospholipid composition.

**Figure 5:**
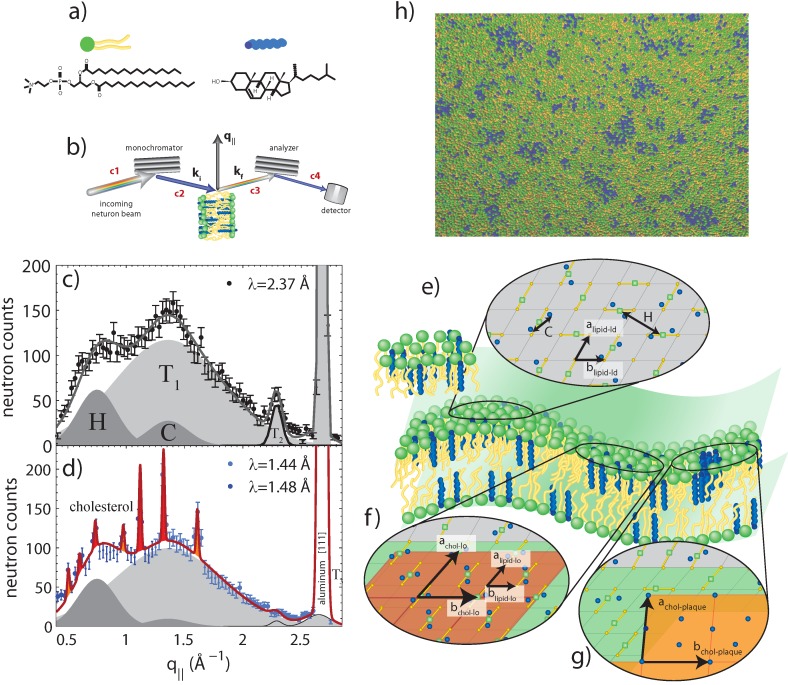
Neutron diffraction of binary DPPC-cholesterol (32.5 mol%) with the cholesterol molecules deuterated [34]: (**a**) schematics of DPPC and (deuterated) cholesterol molecules; (**b**) Sketch showing scattering geometry. q${}_{\left|\right|}$ denotes the in-plane component of the scattering vector; (**c**) Diffraction measured with a high neutron wavelength and low spatial resolution. Broad and fluid peaks are observed, indicative of disordered structures; (**d**) Data measured at low neutron wavelength and high spatial resolution. Sharp peaks are observed, indicative of ordered cholesterol phases coexisting with the disordered lipids; (**e**) Illustration of the different molecular structures: pairs of cholesterol molecules in the L${}_{d}$ regions of the membrane in equilibrium with highly-ordered cholesterol structures, such as L${}_{o}$ domains (rafts); (**f**) and cholesterol plaques (**g**). Simulations also performed in [34] suggest the existence of heterogeneous raft structures, as highlighted in (**h**).

Armstrong *et al.* [33] and Toppozini *et al.* [34] presented the first experimental proof for the existence of highly-ordered lipid domains, induced by cholesterol, within the $L_{o}$ phase of binary phospholipid:cholesterol membranes. Using the coherent properties of the neutron beam, the authors were able to unambiguously resolve signals of $L_{o}$ domains coexisting with $L_{d}$ regions and to determine the molecular structure of lipid and cholesterol molecules in these rafts. Three structures were observed [34]: (1) a fluid-like structure with strongly-bound pairs of cholesterol molecules as a manifestation of the liquid-disordered ($L_{d}$) phase; (2) a highly-ordered lipid-cholesterol phase, where the lipid-cholesterol complexes condense in a monoclinic structure; and (3) triclinic cholesterol plaques, *i.e.*, cholesterol bilayers coexisting of the lamellar lipid membranes. These structures are depicted in Figure 5. The lipid molecules in these rafts were found to form a well-ordered gel phase [33].

In single phospholipid systems, the $L_{o}$ phase was previously believed to be a homogeneous phase. Armstrong *et al.* also determined the dynamic properties that cholesterol imposes on the $L_{o}$ phase and, interestingly, did so before the formal observation of these domains [35,36]. The nanoscale dynamics $L_{o}$ were observed using inelastic neutron scattering, which does not rely on the use of bulky and perturbing probes. The domains in the cholesterol-induced $L_{o}$ phase appeared softer than the $L_{d}$ phase, with a reduced membrane viscosity, but were more ordered than the lipid gel phase.

These studies, for the first time, give a detailed molecular picture of the fluid structure of model lipid membranes. Cholesterol leads to the formation of ordered patches, which are enriched with cholesterol and characterised by drastically different properties, as compared to the surrounding membrane environment. This change in membrane homoeostasis has been shown to lead to reduced health in individuals with high cholesterol. Some of the effects of reduced health include high blood pressure and hypertension, which increases the risk for ischemic heart disease.

## 4. Aspirin

A common treatment for the prevention of ischemia-related events in individuals with increased cholesterol levels is the daily intake of a low-dose of acetylsalicylic acid (aspirin) [37,38].

Aspirin has long been associated with specific interactions when introduced into the body. Aspirin interacts with the cyclooxygenase (COX) pathway, inhibiting platelet aggregation [39]. In patients with high cholesterol, a reduction in platelet aggregation can decrease the incidence of blocked arteries and reduce the chance of myocardial events [40]. This was long been believed to explain low-dose aspirin therapy. Recently, the role of the COX pathway in low-dose aspirin therapy has been called into question, given the growing awareness of so-called “aspirin resistance” [41]. Platelets from aspirin-resistant patients often appear unaffected by the drug, likely through COX-independent mechanisms [42]. The confusion surrounding aspirin has been recently discussed in the media [43].

At the same time, there is increasing evidence for a role of the lipid membrane structure and composition in platelet function [44]. Aspirin has recently been shown to strongly interact with membranes, both biological and synthetic, residing in the lipid headgroup region ([45,46]). In particular, when introduced in model membranes, aspirin has been shown to dissolve harmful cholesterol plaques, leading to a more fluid, healthy bilayer [47]. In addition, aspirin is believed to interact with the membranes of red blood cells, making them more fluid and compressible, which could allow them to flow past barriers with greater ease [48].

**Figure 6:**
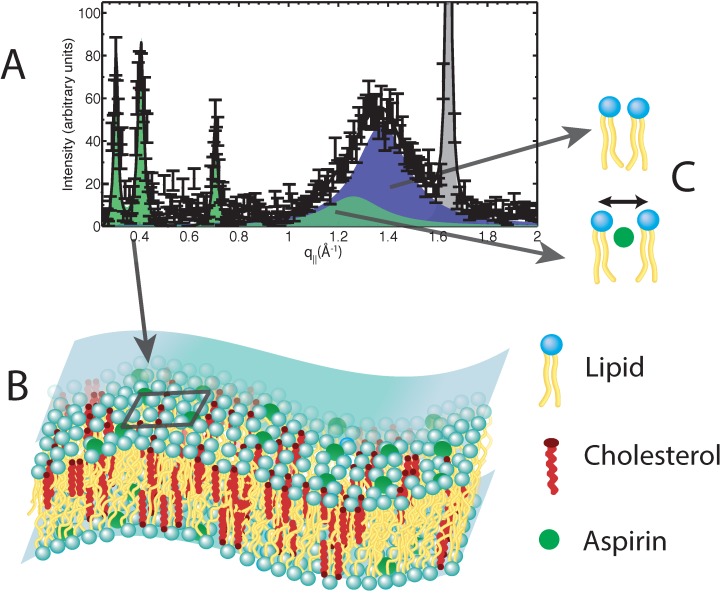
(**A**) Neutron diffraction data obtained from model membranes containing cholesterol and aspirin; (**B**) a 3D cartoon of a membrane containing cholesterol and aspirin, as determined by the neutron data. The cartoon highlights the regular distribution of aspirin (dark square on the membrane), leading to the frustration of lipid raft structures; (**C**) Cartoons highlighting the altered lipid environments introduced by aspirin. Aspirin interacts with the lipid headgroups, leading to an increase in lipid tail separation and an increase in lipid fluidity.

Alsop *et al.* recently performed neutron diffraction experiments on model membranes containing cholesterol and aspirin [49]. The data suggest that aspirin locally alters the lipid environment when introduced into membranes. By interacting with lipid headgroups, aspirin is able to increase lipid fluidity and compressibility, opposing the effect of cholesterol. By working against the effects of cholesterol, aspirin is able to frustrate the formation of lipid domains, fundamentally changing the membrane’s structure and organization. Using the coherence length-dependent neutron diffraction technique, Alsop *et al.* were able to well resolve the nano-scale changes in the lipid structure induced by aspirin. Neutron diffraction gives unprecedented details of the molecular organization in membranes and enables one to develop molecular models, as shown in Figure 6.

## 5. Vitamin E

There is no clear evidence for the health benefits of supplementing our diets with additional vitamin E (*α*-tocopherol), except for specific deficiency syndromes [50]. This is true whether for general heart health or as part of conventional treatments of conditions, such as ischemia-reperfusion injury. This is despite the case of myocardial ischemia reperfusion injury, where maintaining redox homoeostasis is pivotal in the survival of victims [51].

Tocopherol pretreatment is often used to prevent myocardial ischemia reperfusion injury in the case of bypass surgery patients [6]. However, different studies examining the benefits of tocopherol pretreatment yield contradictory results [6,52]. However, a clear molecular mechanism of vitamin E antioxidant action in a cellular membrane is still missing or if such an antioxidant action exists *in vivo* at all. This is especially true when considering the conflicting data in the literature. For example, some argue that it functions as an antioxidant, while others argue from the same evidence that it has some other, not yet identified task. For example, Traber and Atkinson write: “…all of the observations concerning the *in vivo* mechanism of action of *α*-tocopherol result from its role as a potent lipid-soluble antioxidant.” [53]. However, in the same journal issue, Azzi takes the counter argument that “… *α*-tocopherol is not able, at physiological concentrations, to protect against oxidant-induced damage…” [54].

**Figure 7:**
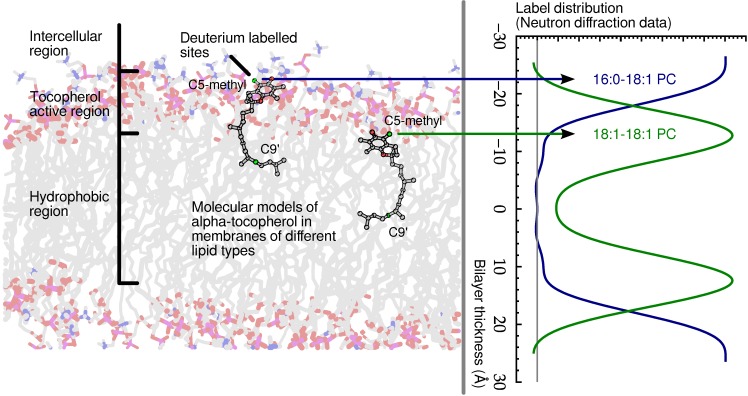
Schematic of *α*-tocopherol in a model lipid membrane as determined by neutron diffraction. The zone of *α*-tocopherol antioxidant action is confined to the region of the glycerol ester and above, extending practically to the aqueous phase. Although *α*-tocopherol can either terminate a lipid radical or intercept diffusing reactive oxygen species, its different locations within bilayers correlate well with its primary activity.

Recently, evidence of an antioxidant mechanism for *α*-tocopherol was presented, which correlates strongly with its physical location in a model lipid bilayer [55]. The data addressed the overlooked problem of the physical distance between the vitamin’s reducing hydrogen and lipid acyl chain radicals. Combined data from neutron diffraction, nuclear magnetic resonance (NMR) spectroscopy and ultraviolet (UV) spectroscopy studies suggested that a reduction of reactive oxygen species and lipid radicals occurs, specifically at the membrane’s hydrophobic-hydrophilic interface, as shown in Figure 7. Such a conclusion has eluded scientists for decades, because no one had yet determined the location of *α*-tocopherol with precision until we applied neutron diffraction with deuterium labelling.

A follow up study determined, by means of small angle neutron diffraction, that not only is *α*-tocopherol’s hydroxyl group located high in the model membrane, but its tail also resides far from the centre of bilayers of 1-palmitoyl-2-oleoyl-sn-glycero-3-phosphocholine (POPC) [56]. In addition, Marquardt *et al.* located the hydroxyl group of *α*-tocopherol above the lipid backbone in 1-palmitoyl-2-oleoyl-sn-glycero-3-phosphoethanolamine (POPE), 1-palmitoyl-2-oleoyl-sn-glycero-3-phospho-L-serine (POPS) and sphingomyelin, suggesting that *α*-tocopherol’s location near the lipid-water interface may be a universal property of the vitamin [56].

Another important result that originated from thermal neutron scattering was determining the location of vitamin E in the prototypical lipid dimyristoyl-phosphatidylcholine (DMPC). Without exception, the data point to *α*-tocopherol’s active chromanol moiety residing deep in the hydrophobic core of DMPC bilayers, a location that is in stark contrast to *α*-tocopherol’s location in other lipids. The discovery of *α*-tocopherol’s residence in the centre of a DMPC bilayer explains some of the conflicting and inexplicable data found in the literature regarding *α*-tocopherol’s behaviour in DMPC bilayers *versus* other phospholipid bilayers [57].

## 6. Concluding Remarks

Neutron beams have become an indispensable tool for cutting-edge research in molecular biology and pharmaceutical sciences. Biological-themed research remains a small and slowly growing component of the science conducted at neutron beam facilities. Annual report data from the Institut Laue-Langevin (Grenoble, France) tracks growth in experimental proposals classified as “biology” from 6% in 2002 to 10% in 2013. However, many experiments classified as “soft condensed matter” often have applications in biochemistry and molecular biology, and including these, as many as one in eight instrument-days at the Institut Laue-Langevin (ILL) is devoted to science involving some biologically-related material.

The study of membranes and membrane-bound molecules requires precise conditions, such as temperature, pressure and hydration, to simulate physiological conditions. Neutrons provide a valuable method for examining these systems, because certain materials, such as aluminium, are transparent to neutrons, allowing for sample environments to be easily constructed and implemented. Neutron scattering studies of biologically-relevant samples have an intrinsic advantage due to the abundance of hydrogen (${}^{1}$H) atoms that can be replaced (labelled) by deuterium (${}^{2}$H). The substitution of deuterium atoms for hydrogen, at selective locations, provides a contrast between two different samples (“labelled” and “unlabelled”). The difference in scattering length density between the labelled and unlabelled sample yields the exact location and distribution of the ${}^{2}$H label. The power of neutrons has been utilized in an attempt to solve many pharmacological and epidemiological mysteries by approaching the problems with a “first principles” molecular mechanism lens.

Deuterium plays an important role in neutron scattering for biology, and to that end, many neutron beam laboratories have established their own ancillary laboratories dedicated to the incorporation of deuterium into biological molecules and systems. For example, the Center for Structural Molecular Biology at the Oak Ridge National Laboratories (utilizing the High Flux Isotope Reactor) established the Bio-Deuteration Lab for this express purpose. The European Photon and Neutron Campus (EPN-campus), home of the Institut Laue-Langevin reactor, now shares its grounds with the Institut Biologie Structurale and, through the Partnership for Structural Biology, has established the Deuteration Laboratory platform (D-LAB).

Most of these experiments are guided by physics-trained biophysicists, working in collaboration with colleagues from biochemistry and biology departments. Ultimately, it will be up to these biologists to flesh-out the theories necessary to reach clinical application. Insights such as those shown above afford physiologists a molecular picture otherwise unattainable without the use of neutron radiation.
